# Understanding School Presenteeism and Absence in Adolescents Affected by Chronic Musculoskeletal Pain: A Qualitative Study

**DOI:** 10.1111/cch.70140

**Published:** 2025-07-15

**Authors:** Beau Sherwood, Lisa Roberts, Rhiannon Joslin

**Affiliations:** ^1^ School of Health Sciences University of Southampton Southampton UK; ^2^ Therapy Services Department University Hospital Southampton NHS Foundation Trust Southampton UK; ^3^ Women's and Children's Department University Hospitals Sussex, St. Richards Hospital Chichester UK

**Keywords:** adolescent, chronic pain, musculoskeletal pain, parent, presenteeism, qualitative absence, school

## Abstract

**Background and Objectives:**

Absenteeism among adolescents experiencing chronic pain is a critical issue, and factors influencing absence have been identified as an evidence gap. Existing research overlooks aspects beyond attendance, such as presenteeism, reflecting adolescents' school functioning before becoming absent. This novel qualitative study sought to identify the influencing factors on presenteeism and absence from school in adolescents (11–18 years) experiencing chronic pain, through exploration of adolescent and parent perspectives.

**Methods:**

This study conducted a secondary analysis of qualitative data using semi‐structured interviews and a timeline drawing from 21 adolescents treated for chronic musculoskeletal pain and 21 parents. Data regarding school experiences were extracted and analysed using the six stages of thematic analysis, identified by Braun and Clarke. Initial codes were identified manually, discussed and validated by all authors in a face‐to‐face meeting.

**Results:**

Thematic analysis of the data identified three themes: (1) understanding the unseen struggle, (2) the feeling of belonging and (3) navigating transitions.

**Conclusion:**

A key finding was the importance of adolescents feeling understood and supported by their peers and teachers throughout their education. Receiving validation of their symptoms, despite the invisible nature of chronic pain, affirmed a sense of security at school, contributed to a more positive school experience and improved adolescents' reported attendance. This has important practice implications in healthcare and education, in validating the adolescents' struggle, fostering a sense of belonging through shared goal setting and advocating for their voices to be heard.

## Summary

- When adolescents experiencing chronic pain perceive school staff and peers as supportive, they report being more likely to attend school.
- Having a designated trusted adult within the school environment was key to supporting adolescents experiencing chronic musculoskeletal pain to function in school.
- Healthcare professionals should carefully consider the prescriptions of walking aids because they can inadvertently contribute to stigma and social isolation.
- There is a need to increase health professional awareness of the importance of school transitions, as these pivotal periods may trigger student absenteeism whilst also providing unique opportunities for re‐engagement in education.
- Healthcare professionals should prioritise school attendance by setting school‐related goals, providing additional support around transition points and adjusting appointment schedules to avoid adolescents missing school.

## 1 Introduction

As per Article 28 in the United Nations (1989) Convention on the Rights of the Child, all children (every human being below 18 years old) have a right to education. Childhood is a critical developmental period in which children can self‐construct an identity, and in a dynamic world of growing complexity, school attendance becomes a pivotal foundation for their future prospects, academic achievement, well‐being and wider development (Enderle et al. 2024). Absenteeism is broadly defined as when a child does not attend school, college or education provision for any reason (Kearney and Silverman 1996), and presenteeism is defined as ‘when a child attends school for any period whilst unwell’ (Woodland et al. 2023, 1). Presenteeism appreciates that adolescents can be present in class without fully participating due to their health (Ferracini et al. 2014).

Musculoskeletal pain is one of the most prevalent causes of chronic pain affecting 25.7% of children and adolescents (Chambers et al. 2024). Cross‐sectional community surveys (Groenewald et al. 2019; Jones et al. 2004; Vervoort et al. 2014), clinical cohorts (Kashikar‐Zuck et al. 2010; Logan et al. 2008; Owiredua et al. 2023) and a systematic review (Norton and Southon 2021) have established that school attendance is adversely affected when adolescents are experiencing chronic musculoskeletal pain. School attendance is a recommended outcome measure for paediatric chronic pain trials (Palermo et al. 2021); however, there is substantial heterogeneity in the way this outcome is reported (Claus et al. 2022; Hechler et al. 2015). Another limitation of measuring school attendance is that physical presence at school does not always mean a child is socially or cognitively present (Gorodzinsky et al. 2011). In addition, Kashikar‐Zuck et al. (2010) found over 12% of adolescents experiencing chronic musculoskeletal pain were homeschooled (Kashikar‐Zuck et al. 2010) and may be missed from data collection as not enrolled within school systems.

In comparison to school absence, there is less research that explores presenteeism and how adolescents affected by chronic pain function at school (Jones et al. 2018). Adolescents experiencing chronic pain within school samples in New Zealand (Farrant et al. 2023) and a Flemish‐speaking area of Belgium (Vervoort et al. 2014) reported poorer outcomes of school functioning across indices of school‐related pressure and satisfaction and bullying (Vervoort et al. 2014). Cognitively, there is less evidence that academic performance is affected (Logan et al. 2008; Mullen et al. 2025; Vervoort et al. 2014) whereas executive functioning did show impairment in adolescents with chronic pain in comparison to healthy peers (Jastrowski Mano et al. 2020). Chronic pain was also found to negatively impact the likelihood of obtaining qualifications associated with pursuing higher education (Mullen et al. 2025). Physically, musculoskeletal pain impairs mobility (Phillips et al. 2025) affecting adolescents' ability to move between classes and preventing school recreational and sport participation (Pourbordbari et al. 2022). Although the school environment is primarily considered in a social context, the emotional and physical demands of school should not be underestimated. The biopsychosocial model (Engel 1977) addresses the requirement for a heuristic approach to understanding and treating chronic pain (Gatchel et al. 2007), and in adolescence, education is a critical consideration.

Logan et al. (2008) emphasised the importance of broadly evaluating school functioning and that poor school functioning before school absence has been linked to presenteeism. This idea is consistent with occupational research featuring a relationship between chronic pain and reduced professional fulfilment (Adams and Salomons 2021). In comparison to adults without pain, those experiencing chronic pain were 64% less likely to report their job as rewarding, 30% more likely to report poor supervisor support, and 28% more likely to perceive discrimination (Adams and Salomons 2021). These psychosocial factors negatively impacted workplace productivity, subsequently leading to burnout (Demerouti et al. 2009). There is evidence that adolescents affected by chronic pain can be confronted with similar challenges to adults, with higher levels of victimisation, fewer friends and being less well‐liked than healthy peers (Forgeron et al. 2010; Greco et al. 2007; Hjern et al. 2008; Kashikar‐Zuck et al. 2007). Simons et al. (2010) discovered that adolescent social functioning mediated the relationship between chronic pain experience and school impairment and highlighted the importance of adolescents maintaining social connections at school. However, it remains unclear what underlying mechanisms impair school attendance and functioning for adolescents experiencing chronic musculoskeletal pain.

Understanding the factors influencing both absenteeism and presenteeism is currently an evidence gap and requires knowledge of the experiences of adolescents affected by chronic musculoskeletal pain and the experiences of their parents to develop future strategies to support adolescents in school, promote engagement in education, reduce absenteeism and build the capacity of the future workforce. To address this knowledge gap, this UK study explored the influence of chronic musculoskeletal pain on school presenteeism and absence, addressing the following research question: What factors influence presenteeism and absence from school in adolescents experiencing chronic musculoskeletal pain?

## 2 Methods

### 2.1 Study Design

This qualitative study comprised a secondary analysis of qualitative data from a primary research study (Joslin et al. 2021, 2023) that was informed by Q methodology (Brown 1993, 1996; Stephenson 1993). Qualitative data collected from interviews in the primary study facilitated a broader use of information to explore a novel research question regarding school attendance and presenteeism that was beyond the scope of the primary study and maximised data utility in this under‐represented population (Tate and Happ 2018). Secondary qualitative analysis is a known methodology and is most frequently used when one or more authors have been involved in the primary research (Heaton 2008). Ethical approval for the secondary analysis was granted through the University of Southampton Ethics Committee (ERGO No.: 82220). The consolidated criteria for reporting qualitative research (COREQ) guidelines have been used to ensure rigor in the analysis and reporting (Tong et al. 2007).

### 2.2 Participant Recruitment

Recruitment took place at two hospital sites in England. Participants were recruited via local services (i.e., physiotherapy outpatient departments, rheumatology clinics and psychology services) and a tertiary multidisciplinary chronic pain service. This multifaceted approach aimed to capture a diverse data set including adolescents at different stages of their treatment. The inclusion criteria for the primary study were adolescents aged 11–18 years experiencing chronic musculoskeletal pain and/or their parent(s). Chronic musculoskeletal pain was defined as pain presenting for more than 3 months located over joints, muscles or soft tissues. The exclusion criteria included adolescents with systemic disease (e.g., juvenile idiopathic arthritis) or long‐term comorbidities causing permanent disability and those unable to express their views either verbally or in written format in English. No further additions to the primary research inclusion and exclusion criteria were required for this secondary analysis.

Clinical staff initially pre‐screened and invited all eligible adolescents and parents by mail. Adolescents were then purposively sampled based on their age, sex and treatment stage. Once a month, the researcher updated clinical staff on outstanding gaps in the recruitment sampling grid, attending clinics that staff thought were potentially advantageous for recruiting missing viewpoints. Clinical staff gave eligible participants the study information during their appointment (face‐to‐face), and the researcher was present in a separate room. Adolescents and parents were offered the opportunity to discuss the study with the researcher. All participants had the study information for over 48 h prior to taking part and were free to withdraw from the study at any point, without prejudice. All parents and adolescents who wanted to take part were recruited. The sample size for the primary study was estimated to be 18–24 interviews with adolescents and 18–24 interviews with parents. Interviews were stopped when no new initial codes were identified in the last three interviews, and the research team felt the data had reached a point where further interviews would not add to the overall story.

### 2.3 Data Collection

Semi‐structured interviews were completed via multiple media (face‐to‐face at home or in hospital, video or telephone call, or an instant messaging platform), as chosen by participants. Additionally, participants completed a timeline drawing of their treatment journey during their interview (Adriansen 2012; Hurtubise and Joslin 2023). Where possible, adolescents spoke without parents present and parents spoke without their child present. The interview schedule (Appendix 0) was adapted by two patient representatives using the timeline instructions from Save the Children Norway (2008) as a template. The final author (R.J.), a qualified physiotherapist with experience working with this population of children, conducted all the interviews. Children were recruited from hospital sites where R.J. had not been involved in clinical care, participants were unknown to her and she was introduced as a researcher. All interviews were audio‐recorded and transcribed verbatim. Field notes and reflections were made immediately and used to contextualise the transcripts and analysis, when necessary.

Adolescents and parents provided written consent to participate. For children aged 11–16 years old, their parents had to be aware of their participation to comply with ethical approval. All participants kept their timeline drawings, and photographs of the drawings were provided for analysis. Transcripts were not returned to participants for member checking. To ensure confidentiality, interview transcripts and timelines containing identifiable information were replaced with numerical codes of unknown correlation.

### 2.4 Data Analysis

All 42 interview transcripts (21 adolescents and 21 parents) and corresponding timelines from the primary study were analysed in this secondary analysis because education was discussed within all interviews. The six stages of reflexive thematic analysis were used to develop themes: (1) data familiarisation; (2) systematic data coding; (3) theme generation; (4) theme review; (5) definition, refinement and naming of themes; and (6) report writing (Braun and Clarke 2006, 2019, 2022). Data analysis from the interviews and transcripts was completed manually, and the timeline drawings were instrumental in assessing changes in school attendance and the transition from presence to absence or vice versa and the causes for change. The main author (B.S.) coded the timeline drawings and transcripts line by line. Quotes related to school and education were highlighted and moved into a Microsoft Excel Spreadsheet, with each entry labelled according to the child's age, school year and attendance status. Initial codes were selected and grouped based on identified patterns and moved into Microsoft Word. A subsequent 3‐h data analysis session with all authors involved discussion and agreement on the initial codes and development of themes. This process involved using mind maps on a large whiteboard and then rearranging the codes to represent key themes. Authors R.J. and L.R. had been involved in coding the transcripts in the primary study and therefore were familiar with the content. The themes were then revisited as the report was written and edited. To enhance trustworthiness, credibility was established through detailed discussions between authors, documentation of the audit trail, triangulation using different methods (interview transcript and timeline), and inclusion of quotes across the sample (Noble and Smith 2015). As this was a secondary analysis, the decision to stop data collection was established a priori in the primary study when no new codes were established in the final three interviews.

## 3 Results

Table 1 shows adolescents' demographic information. All adolescents (*n* = 21) were enrolled in secondary education or higher at the time of their interview; however, 10 adolescents (48%) reported the onset of pain during primary school. Three adolescents (14%) were pursuing further education. Forty‐two interviews (21 adolescents and 21 parents) provided an understanding of 24 narratives of school experience. The 24 narratives were reported from different perspectives: Six were from the adolescents' perspective only, three were from the parents' perspective only and 12 narratives were from multiple perspectives including the adolescent and one parent (*n* = 10) or the adolescent and both parents (*n* = 2). One adolescent and parent chose to be interviewed together, and the remaining interviews were completed separately. The parents included four fathers and 17 mothers. The length of the interviews ranged from 28 to 98 min.

**TABLE 1:** Demographic data of the participants (*n* = 21).

Characteristic	Results
Gender, *n* (%)	
Female	18 (86%)
Male	3 (14%)
Age in years (mean, range)	14.8 (11.11–18.0)
School year (mean, range)	9 (7–13)
Place of education, *n* (%)	
Secondary school	16 (76%)
College	3 (14%)
Hospital school	1 (5%)
Home school	1 (5%)
Duration of pain in months (mean, range)	40 (5–108)

Table 2 shows the three themes developed from reflexive thematic analysis. The themes elucidate adolescents' school experience and the factors influencing presenteeism and absence.

**TABLE 2:** Themes, a summary and example quote(s).

Theme	Summary	Example quote(s)
Understanding the unseen struggle	Feeling genuinely understood and supported by school staff and peers had the potential to increase school attendance, whereas feeling unsupported and disbelieved had the opposite effect.	Mother 6: ‘I think maybe if she had just had like one teacher or someone who actually supported her through the whole thing, other than just being me, then she might have been a bit more, her attitude towards school and everything, might be a bit more positive’.
The feeling of belonging	Social connection and acceptance within the school environment appeared to positively reflect attendance, whilst exclusion, stigma and bullying contributed to absenteeism.	Girl 19: ‘… so it's very hard socially to talk to people because everyone knows me as cripple for some reason’. Mother 19: ‘I think part of the problem is that … when they were at school there will have been kids that were making her feel like bullied’.
Navigating transitions	Key school transitions could either support or hinder school engagement and attendance, either facilitating renewed connections and motivation or exacerbating vulnerability and isolation.	Mother 15: ‘… when she went back [to school] she said “I felt I had to get know my class all over again and work out my place within the class”’.

### 3.1 Theme 1: Understanding the Unseen Struggle

This theme describes the disconnect between the outward appearance and internal struggle of adolescents experiencing chronic pain, leaving many adolescents feeling isolated and unsupported at school. If an adolescent felt supported at school by staff and other students, they appeared to attend and engage at school, whereas if they felt disbelieved and unsupported, they reported disliking school and were more likely to be absent. Adolescents did not feel believed by many staff, including the school nurse whom they hoped would understand.

The woman [school nurse] said jokingly ‘she's a difficult one she is’ she meant it in a joking way as if to say there's not a lot you can do type of thing but [my child] said to me ‘I thought she [the school nurse] understood me, but she doesn't, even she doesn't believe me’ and I was like it's not that no one believes you it's hard for people to understand. (Mother 13)

The invisibility of chronic pain led to the dismissal of its severity. When no visible sign of injury or illness was present, adolescents reported that teachers and students assumed their problems were resolved. They were expected to take part in school activities, including physical education (PE). Adolescents and parents faced scrutiny from teachers and administration staff who questioned regular absences, and parents were asked to provide proof in the form of a formal diagnosis. Pressure from school to provide evidence from the hospital appeared to be driven by the prioritisation of school attendance; however, adolescents reported this overlooked their ability to participate and engage.

…she [my daughter] says ‘no, cause once I'm there they won't let me go [home] because of attendance’ and she gets quite annoyed about it, that it's like well, they don't care even if I'm doing anything it's literally just physically being there in the building so they can put a tick next to my name. (Mother 19)

Adolescents found themselves caught in a vicious cycle where their symptoms fluctuate unpredictably, resulting in difficulty concentrating in class and frequent absences due to hospital appointments. The inability to engage in class alongside their peers often felt punitive. Adolescents described feeling marginalised such as being isolated into areas with children who misbehaved (‘the naughty kids’) or spending their days alone in the library. As a result, one family reported homeschooling their child. Furthermore, parents perceived that in the absence of a diagnosis, their adolescents were denied support provisions or access measures from the school and were unable to navigate the school environment independently. Most adolescents frequently reported challenges with PE; however, there was one young person whose PE experience was described positively and appeared to be associated with remaining in school.

Yeah but my teacher was really understanding, he was really nice and he basically like helped me set up, I was a coach, I taught people how to do some stuff … One it stops immense boredom and two … I can help myself by not making too much pain for me, but also I can help other people by teaching them how to do things, coaching them and that's when I can join in, it's like an element of fun by joining in [sic]. (Girl 20)

Conversely, feeling excluded and having to sit and watch their peers take part led to adolescents feeling sad and frustrated. One adolescent reported that school staff stopped them from taking part in PE due to frequent joint dislocations. Watching everyone doing something they loved and not being able to take part had psychological consequences. One adolescent reported that watching PE made them the lowest in mood that they had ever been. These experiences built resentment towards both the school and teachers as a result. Adolescents felt more at ease attending school when teachers and peers acknowledged their difficulties and expressed empathy for their experiences. When parents worked with teachers and suitable arrangements were established, such as a reduced timetable or modified curriculum, adolescents reported improvements in attendance.

### 3.2 Theme 2: The Feeling of Belonging

The school environment offered opportunities for connection and belonging that were associated with adolescents attending school. On the contrary, social challenges particularly involving friendships were reported as contributors to absence. Adolescents highlighted that making and maintaining friendships was hard and there was an emotional strain associated with broken friendships. Indeed, some attributed physical limitations to friendship problems. Some parents reported that their child withdrew and disconnected from their friends, whilst other parents described occasions where their child's friends failed to understand chronic pain and did not accommodate them. In addition to the challenges of forging friendships, adolescents expressed feeling estranged from their school community. Some adolescents were cautious of attending school and walking in the corridors, fearing exacerbation of their pain from accidental collisions with others. To reduce being victimised by students who singled them out and called them *faker* or *cripple*, participants attempted to validate their invisible pain.

He started senior school and then because he was concerned about not having a diagnosis and struggling and people not really knowing why he was walking badly, that's when he started really using crutches at school. (Father 20)

However, using a mobility aid was reported by other adolescents to draw unwanted attention, causing others to view them as different, which consequently led to feelings of inadequacy compared to their peers. Additionally, a lack of understanding among peers left participants vulnerable to bullying. One adolescent reflected on the contrast in their experience between primary and secondary school, with students taking advantage of the larger and less supervised environment of secondary school. A group of adolescents described victimisation and bullying at school exacerbated by the use of crutches or a wheelchair, which improved once the adolescent was walking unaided, the same as everyone else.

People came up behind me and started pushing me about, I got tipped out of it [wheelchair] once and I had to pick it up myself and try and put myself back in it, dragging my legs across the floor which was very embarrassing in front of people. (Girl 19)

Adolescents experienced stigma surrounding chronic pain, including scepticism from peers, other students and teachers that resulted in adolescents feeling isolated. Adolescents struggled to reconcile their own experiences with societal expectations of *normal* and attempted to become invisible at school. When their peer group did engage with them, adolescents feared rejection and perceived themselves as boring or a burden, which could lead to self‐isolation. Conversely, trusted friendships could provide adolescents with a sense of belonging and were consistently associated with the desire to attend school. These friendships affirmed adolescents' values, and they felt accepted as they were.

It basically makes me feel that people still like me for the way I am even though I don't necessarily like the way I am at the moment [sic]. (Girl 02)

The support from friends provided mental relief, and reassuring adolescents emotionally helped alleviate feelings of loneliness and fostered a sense of security. For some, the impact of empathy from peers who share similar experiences was evident. Friendships served as a positive distraction, diverting attention away from pain and allowing participants to focus on their friends instead.

### 3.3 Theme 3: Navigating Transitions

Transitions reported by adolescents and parents included starting a new academic year, moving to a new school (including transitioning from primary to secondary school or secondary school to college or moving from one school to another) and returning to school after an extended period of absence. The transition from primary to secondary school was the most frequently reported. All transitions could improve or worsen adolescents' school attendance and engagement.

Most adolescents described a negative response from changing school, moving from a school where they felt settled to one where they felt vulnerable. Beyond navigating an unfamiliar environment, an increase in physical demands such as having to walk further and carry their bag often added to the complexities of their condition. Additionally, a shift in social networks left adolescents exposed to interrogation from school staff unfamiliar with the adolescent's circumstances.

Changing friends as well, he had some friends going to it but again some friends not so losing a bit of support there in the school setting and then obviously having to travel around from classroom to classroom and all of that [sic]. (Father 20)

Conversely, for a few adolescents, a new school offered a fresh start and the opportunity to establish new friendships. They described finally finding a group of people that were like them and so felt like they ‘fitted in’.

When adolescents had extended absences from school and attempted to return, this was overwhelmingly described negatively. On return from prolonged absence, adolescents reported they were detached from school, socially isolated and apprehensive about reintegrating into the classroom environment. Parents also acknowledged this social isolation.

I was kind of concerned that when she went back into the classroom she would kind of feel like a bit of an outsider, didn't know her place within the class. (Mother 11)

A few parents also voiced concerns that their child's pain‐related absence might be masking anxiety akin to starting school for the first time. Furthermore, parents of previously high‐achieving adolescents expressed anxiety related to their child's academic performance and the pressure to maintain their academic standing. To re‐engage in school, adolescents became more reliant on parents for support. Many parents, especially mothers, described rearranging their lives to drive their child to school, whereas previously the young person had used public transport or a school bus or walked.

The transition between school years and returning to school after a summer break could improve or worsen school attendance and engagement. For some adolescents, the desire to pass exams, secure employment, and avoid reliance on welfare benefits became a driver to improve school attendance. This was most apparent in the final year when exams took place. However, the increased pressure from exams had the reverse effect on some adolescents. Similarly, some parents portrayed a sense of disappointment regarding their child's reduced expectation to secure employment, whereas other parents recognised their child tended to overwork academically. Finally, interactions with healthcare professionals gave a few adolescents a desire to engage in education and pursue a career motivated by their own experiences.

She has also been talking about, I mean early stages in her life, but careers and one of the things she does talk to us about is becoming a nurse … cause maybe what's she's been through, she doesn't want it to happen to others and she's recognised herself how people have spoken to her, but who knows? (Father 17)

## 4 Discussion

Through the lived experiences of adolescents and parents treated for chronic musculoskeletal pain, key factors were identified that influenced presenteeism and school absences. These included the support of school staff, peers and students; having a sense of belonging; and being able to navigate school transitions.

Firstly, when adolescents perceived school staff and students as unsupportive and dismissive of pain, they appeared less likely to attend school. Hjern et al. (2008) found that being treated poorly by teachers and harassment by peers were factors associated with the presence of chronic headache and abdominal pain; however, this was not explored in terms of school absence. Aligning with the current study findings that a lack of a formal diagnosis intensified the challenge of adolescents receiving validation and accessing support (Simons et al. 2012), it was found that adolescents withheld a strong desire to legitimise the physical nature of their pain to others, particularly when it has been implied that the pain is not real. There are known challenges to school staff understanding adolescents' experiences with chronic pain, including lack of time and opportunity, and not having methods and structures to establish the cause of pain (Golsäter et al. 2019). In Sweden, a school‐based intervention delivered by school nurses for adolescents experiencing chronic pain found that the trust between the adolescent and school nurse was fundamental to how a child managed their chronic pain at school (Wallbing et al. 2023). A child perceiving they had a teacher who cared moderated the relationship between perceived school stress and satisfaction with life (Grasaas et al. 2024) and increased school enjoyment (Children's Commissioner 2024). The current study supports the importance of adolescents having a designated person at school whom they trust, but this was not a school nurse in any of the cases. Trusting an adult at school appears foundational to adolescents with chronic pain regaining their ability to manage their situation and restore their identity (Golsäter et al. 2019; Nilsson et al. 2017; Wallbing et al. 2023). Ideally, healthcare and educational services could work collaboratively to ensure diagnosis understanding and consistent management; however, families not permitting healthcare services to contact their child's school was a barrier to children taking part in an intervention to improve school functioning (Logan and Simons 2010). This wider family perspective needs further exploration if future interventions and guidelines are to be implemented successfully.

Secondly, adolescents achieving a sense of belonging through friendships appeared vital to maintaining school attendance and functioning; yet adolescents and parents described difficulties starting and maintaining friendships. This finding reflected the peer relationship deficiencies known to exist for adolescents affected by chronic pain, including having fewer friends, isolation, victimisation and bullying (Forgeron et al. 2010; Hjern et al. 2008; Kashikar‐Zuck et al. 2007; Mullen et al. 2025; Vervoort et al. 2014). The current study identified that walking aids were associated with peer victimisation, which is a novel finding and may help to explain why adolescents with musculoskeletal pain appear more vulnerable to stigma. A narrative review of pain‐related stigma research and qualitative focus groups (Wakefield et al. 2022; Wakefield et al. 2018) found that peers and school personnel enact pain‐related stigma towards adolescents with chronic pain. As observed by parents in the current study, Forgeron et al. (2011) suggested that when adolescents with chronic pain do not perceive their friends as providing support, they may avoid these social situations. The effects of peer ridicule on self‐esteem and psychological well‐being were, in some cases, significant, contributing to children's decision to remain absent from school. As suggested by Norton and Southon (2021), these findings suggest the need for targeted early intervention to improve adolescents' school functioning prior to them becoming absent, such as interventions to enhance social relationships and schools preventing and addressing victimisation and bullying.

Thirdly, transitions, such as moving to a new academic year or school, were key points in time that could result in adolescents becoming absent or making the decision to return to school after an extended period of absence. Missing a day of school in the first week of term has been shown to predict adolescents' overall absence from school in England, with adolescents being absent in the first week of the term being associated with unauthorised absence of around 35% (Children's Commissioner 2022). During these transition points, parents had an important supporting role. In healthcare transitions from child to adult services, parental support has been recognised (Higginson et al. 2019); however, the role of parents during these educational transitional points requires further research to appreciate the wider family influence for adolescents experiencing chronic pain. Awareness of the importance of transition points for children's attendance within clinical practice is limited, and the opportunity for transition points to facilitate positive change is scarcely explored and warrants further research.

Unique and complex interactions were captured within the themes that crossed physical, psychological and social factors supporting the need for guidelines (Golsäter et al. 2019; Solé et al. 2018) underpinned by theoretical frameworks such as the biopsychosocial model (Engel 1977). This would facilitate personalised and holistic care for children experiencing chronic pain in school (Golsäter et al. 2019; Nilsson et al. 2017; Nilsson et al. 2019).

### 4.1 Clinical Implications

To minimise isolation and victimisation of adolescents with musculoskeletal pain, the current study suggests healthcare professionals need to be aware that the provision of walking aids, splints and wheelchairs can unintentionally contribute to stigma or reinforce negative self‐perceptions, making children feel ‘sick’ or different compared to their peers. Furthermore, their misuse to validate a child's pain creates challenges in removing them. Instead, as per The Royal College of General Practitioners' principles on maximising school attendance (Christmas 2023), healthcare providers could adopt a strengths‐based approach to inclusion, focusing on modifications that encourage ongoing participation. Additionally, by raising awareness of the importance of transition points for children's attendance, healthcare professionals could incorporate school functioning within treatment programmes, for example, set goals within the school environment and proactively schedule appointments before key transition points to build a child's confidence and offer support at these difficult points. School attendance should also be considered in service design, extending hours beyond the school day to avoid adolescents missing school due to hospital appointments.

There are important implications for teachers and school administrators too, which could include exploring the options for a classroom to be accessible without walking aids where possible (e.g., on the ground floor), including the child in PE classes, involving them in coaching, for example. In addition, recognising parents' attempts to minimise school absence with healthcare appointments by avoiding punitive letters and instead discussing with parents and children what can be done to best support them will foster a collaborative and supportive approach to the challenges of living with chronic pain.

### 4.2 Limitations and Future Research

Whilst this novel study included a comprehensive corpus of data from 42 participants, the findings should be interpreted with caution due to several limitations. Firstly, this study involves secondary analysis; therefore, the interview guide was not specifically developed to answer the research question of this study. This was partially mitigated, however, as the authors of the primary study were directly involved in the current study. Nevertheless, we recommend that further primary research should specifically explore adolescents' education experience and include absence metrics. Notably, this study explored accounts from parents and adolescents only. To gain a holistic and comprehensive understanding, future research should incorporate the views of teachers, healthcare professionals and other students. Additionally, the opinions of adolescents represent experiences of education in England and therefore may not reflect the experiences of schools in different countries engaging in different educational models and pathways. It would be beneficial to conduct similar studies in countries with different educational institutions and academic governance to explore issues of convergence and divergence. Lastly, not collecting sample characteristics of ethnicity and socio‐economic status hindered the understanding of sample diversity and representativeness and would need to be captured and described in future work.

## 5 Conclusion

The multifactorial challenges adolescents experiencing chronic musculoskeletal pain faced influenced the bidirectional shifts from absence to presence at school. The current study highlights areas where schools can produce a supportive learning environment nurturing intellectual, social, emotional and physical growth. Adolescents' perspectives underscore the critical importance of feeling understood and supported by teachers and peers throughout their education. However, the influence of schools' support systems is only one part of a wider network of potential support and belonging that bridges home, community and healthcare. Efforts to ensure that adolescents flourish in educational settings despite pain must involve building strong collaborative relationships among adolescents, their parents, schools, community services and healthcare professionals. Constructing such partnerships will likely be a challenging process, but one that will significantly contribute to achieving more positive school experiences and securing improved attendance rates in adolescents experiencing chronic musculoskeletal pain.

This novel study highlights that adolescents experiencing chronic pain require individualised, holistic care that involves collaboration between healthcare providers, designated adults within the school environment and families. It is key for health professionals to judiciously review their provision of walking aids as these can inadvertently contribute to stigma and social isolation and review service provision to potentially adjust appointment schedules to minimise absence from school and support families with the challenges of living with chronic pain.

## Author Contributions

**Beau Marie Sherwood:** conceptualization, formal analysis, investigation, methodology, visualization, writing – original draft, writing – review and editing. **Lisa Roberts:** conceptualization, formal analysis, investigation, methodology, writing – review and editing. **Rhiannon Joslin:** conceptualization, formal analysis, investigation, methodology, writing – original draft, project administration, writing – review and editing.

## Ethics Statement

University of Southampton Ethics approval was obtained for the secondary analysis (Ref: ERGO 82220), and the National Health Service (NHS, Leeds, UK) Research Ethics Committee (Ref: 18/SC/0138) approved the primary programme of research.

## Consent

All participants have given written consent to participate.

## Conflicts of Interest

The authors declare no conflicts of interest.

## Data Availability

Due to ethical restrictions and the sensitive qualitative content, the raw data will not be shared.

## Semi‐structured interview schedule of primary study

**Table:**

Broad question	Probing follow‐up questions
Looking at the empty timeline, can you first go to the ‘START’ point. 1 Tell me about yourself at the start of your treatment when you first came to hospital for pain treatment.	What was your pain like? Where did it affect? What were you able to do? How did you spend your time? What were you saying? How did you feel?
Now go to the ‘END’ point 2 At the end, describe yourself at the end of an ideal treatment when you no longer need to come to hospital (this may be in the future).	What would you be doing? Where would you be spending time? What would you be saying? How would you be feeling? What would your parents and family say?
Joining the dots 3 Now, consider your journey from the start of treatment until the end and join the ‘start’ and ‘end’ dots to show what your journey has looked like so far and what it might look like into the future. 4 Now, mark the line with the point where you are now on this journey, and write ‘I am here’ and an arrow.	Add any important events that have happened or are happening in your life.
Positive signs 5 How would you know a treatment was working? Write positive signs on your treatment timeline	What are the early signs/final signs a treatment is working? What changes make the biggest difference? Do you think health professionals/parents notice these changes? Tell me more about x that you mentioned? Can you tell me more about why you chose these?
Negative signs 6 How would you know a treatment was not working? Write negative signs on your treatment timeline.	What are the early/late signs that tell you treatment is not working? What changes cause the most impact and would stop you continuing the treatment? Do you think health professionals/parents notice these changes? Tell me more about x that you mentioned? Can you tell me more about why you chose these.
Change over time If we spoke to you at the start of treatment, would you have said anything different?	
